# Applying the theory of planned behavior and the technology acceptance model to patient adherence to medical innovations in interventional radiology

**DOI:** 10.1590/0100-3984.2025.0058

**Published:** 2026-02-27

**Authors:** Mateus Picada Correa, Camila Biedler Giordani, Joaquim Mauricio da-Motta-Leal-Filho, Marcos Areas Marques, Jaber Nashat Saleh, George Bedinelli Rossi

**Affiliations:** 1 Instituto Vascular de Passo Fundo (Invasc), Passo Fundo, RS, Brazil.; 2 School of Medicine, Universidade de Passo Fundo, Passo Fundo, RS, Brazil.; 3 School of Medicine, Atitus Educação, Passo Fundo, RS, Brazil.; 4 Institute of Radiology, Universidade de São Paulo, São Paulo, SP, Brazil.; 5 Universidade Federal do Rio de Janeiro, Rio de Janeiro, RJ, Brazil.; 6 Universidade do Estado do Rio de Janeiro, Rio de Janeiro, RJ, Brazil.; 7 School of Arts, Sciences, and Humanities, Universidade de São Paulo, São Paulo, SP, Brazil.

**Keywords:** Vascular surgical procedures, Radiology, interventional, Behavior therapy, Educational technology, Diffusion of innovation, Quality improvement, Procedimentos cirúrgicos vasculares, Radiologia intervencionista, Terapia comportamental, Tecnologia educacional, Difusão de inovações, Melhoria de qualidade

## Abstract

Interventional radiology has advanced significantly in recent decades, enhancing quality of life with treatments that are safer and less invasive. Despite these benefits, public awareness about vascular procedures remains limited, which affects the adoption of these therapeutic interventions by patients and physicians alike. Behavioral science theories, such as the theory of planned behavior (TPB) and the technology acceptance model (TAM), offer a robust framework to address this gap by shaping patient health-seeking behaviors. The TPB examines how attitudes, norms, and perceived control influence behavior, whereas the TAM focuses on technology acceptance based on ease of use and usefulness. Attitudes reflect opinions, subjective norms indicate social pressure, and perceived control relates to confidence in performing the behavior. Applying these models can improve patient acceptance and health care outcomes. Collectively, these models are instrumental frameworks in medical innovation, offering a systematic approach to evaluating and promoting the acceptance of new technologies. As health care continues to evolve, with rapid technological advancements, this narrative review underscores the potential role of these models in facilitating the successful integration of medical innovations, enhancing patient care, and optimizing health care practices.

## Abstract (pt)

A Radiologia Intervencionista avançou significativamente nas últimas décadas, melhorando a qualidade de vida com tratamentos mais seguros e menos invasivos. Apesar destes benefícios, a conscientização sobre os procedimentos vasculares permanece limitada, tanto para os pacientes como pelos médicos, afetando a adoção de dessas terapias. Teorias da ciência comportamental, como a Teoria do Comportamento Planejado (TPB) e o Modelo de Aceitação de Tecnologia (TAM), podem ser usadas para preencher essa lacuna, influenciando nas decisões dos pacientes. O TPB analisa como atitudes, normas e controle percebido influenciam o comportamento, enquanto o TAM foca na aceitação de tecnologias pela facilidade de uso e utilidade. Atitudes refletem opiniões, normas subjetivas indicam pressão social, e controle percebido trata da confiança em realizar o comportamento. A aplicação desses modelos pode melhorar a aceitação do paciente e melhorias em práticas de saúde. Juntos, esses modelos são estruturas operacionais na inovação médica, oferecendo uma abordagem sistemática para avaliar e promover a aceitação de novas tecnologias. À medida que os cuidados de saúde continuam a evoluir com rápidos avanços tecnológicos, essa revisão narrativa revela que a aplicação destes modelos pode ser necessária para facilitar a integração de inovações médicas, otimizando as práticas de saúde.

## INTRODUCTION

Interventional radiology (IR) has improved over the last few decades. The new intervention techniques in pain management, benign diseases, and oncology have improved quality of life with reduced rates of mortality and complications, as well as shortened hospital stays^(1,2)^. Although most physicians are already aware of the benefits of IR in this field, there are still challenges in the general knowledge of the population about the specialty and its therapies, affecting the direct search for its treatments^(3)^.

There are two theories in behavior sciences that are been progressively used in medicine in the last years and can be used to modify patient behavior^(4)^. The theory of planned behavior (TPB) was developed by Icek Ajzen in the late 1980s^(5)^. It is a psychological theory used in order to understand and predict human behavior, particularly in the context of decision making. The TPB posits that intention is the best predictor of behavior. Therefore, individuals who wish to perform a specific behavior are influenced by three main factors: attitudes, subjective norms, and perceived behavioral control. The TPB is widely applied in several areas to analyze and modify behaviors through the manipulation of these determinants. Secondly, the technology acceptance model (TAM), created by Fred Davis in 1989, is a framework that evaluates technology adoption by considering user perceptions of the ease of use and usefulness of the technology in question^(6)^. In medicine, the TAM can assess the acceptance of new interventions by patients and especially by health care professionals.

The TPB is used in order to identify any behavior of the client (in this case, the patient), and the TAM is used in order to identify the strategies that should be employed to promote the adoption of new behaviors.

Understanding patient perceptions can guide the design, implementation, and promotion of innovative medical practices and technologies, ultimately improving health care delivery and patient outcomes^(7)^. This narrative review discusses ways to apply these methodologies to change patient attitudes toward the decision-making process to a wider acceptance of new IR procedures, given that there is often resistance among patients to accepting new technologies^(8)^.

This review was constructed from a non-systematic, exploratory, comprehensive literature search, aiming to identify the most relevant literature regarding the application of the TPB and the TAM to patient adherence to medical innovations in IR. The study was approved by the Research Ethics Committee of the Universidade de Passo Fundo (Reference no. 5.065.505).

## DISCUSSION

This article does not aim to prescribe specific strategies for promoting new interventions, but rather to introduce interventional radiologists to relevant theoretical frameworks capable of facilitating the adoption of innovative procedures and technologies. The application of these theories, alongside the selection of appropriate methodologies, is contingent upon the specific research objective. For example, assessing the knowledge of referring physicians regarding a particular procedure will necessitate a distinct analytical approach compared with evaluating the satisfaction of patients who underwent the same procedure.

### Using the TPB in decision making

As mentioned, the TPB suggests that the interests and behaviors of individuals are influenced by their attitudes, subjective norms, and perceived behavioral control.

The first step in bringing about a paradigm shift should be an assessment of current opinions and attitudes of the target population. One must, therefore, begin to understand current patient attitudes and knowledge regarding IR and surgical procedures. This can be done through surveys, interviews, or focus groups. Correa et al. (unpublished data) assessed the impression of the population of the city of Passo Fundo, Brazil, in relation to physicians in the region. The analysis of those data identified the specific factors and attitudes that positively influence patients’ decision-making processes. These may include the risks, benefits, effectiveness, and comfort considered to be associated with each procedure. The second step is widely used in an isolated manner by caregivers and is called change of attitude. The following strategies are used:

1. Supplying information—Medical education is indispensable in changing decisions. Patients for whom these procedures are indicated should be educated about IR and surgical procedures in general, including their benefits, risks, and success rates, performed in clear and accessible language to ensure patients can make informed decisions.
2. Addressing misconceptions—Any misconceptions or misunderstandings that patients may have about the procedures must be identified and corrected. These misconceptions can be identified in the initial questionnaire, and evidence-based information should be used to support the claims.
3. Highlighting success stories—Stories of patients who underwent successful IR procedures should be shared, with an emphasis on positive experiences and useful results. An honest, non-sensationalized approach to patient outcomes is essential.

The next step refers to changing subjective norms^(4)^. Subjective norms represent the perception of social pressure to perform a behavior, and changing those norms involves influencing how people believe others value and expect that behavior. The following situations must be addressed^(4,7)^:

- Encouraging peer support—Create support or patient groups with others who have undergone similar procedures. Sharing experiences can positively influence perceived measures.
- Involving family and friends—Encourage patients to discuss their options with family and friends who can provide emotional support and help with decision making.
- Providing tools and resources—The perceived behavioral control concerns the individual’s opinion of their ability to perform a given behavior. Offer a range of decision-making tools and resources to help patients understand their options and make informed choices.
- Shared decision making—Collaborate with patients and involve them in the decision-making process. Let them voice their opinions and concerns.
- Ongoing support and monitoring—Provide ongoing support and information as patients progress through the decision-making process. This may include additional consultation, access to relevant materials, and resolution of any new concerns that may arise.

Darker et al.^(9)^ designed the first randomized trial investigating the use of TPB measures in promoting exercise in a general population. They found an increase in perceived behavioral control and that intentions and attitudes became stronger in the group in which the theory was used. Piotnikoff et al.^(10)^used the TPB to identify the perceived behavioral control of obese adolescents in Alberta, Canada, to suggest changes in health programs in the region, as has been done elsewhere with the TAM^(8)^.

### Applying the TAM

Applying the TAM to medical innovation can help ensure that new technologies and practices are embraced by the health care community, ultimately leading to improved patient care and outcomes.

The fact that technology is not widely adopted is due to insufficient understanding or stereotyping of the target segments characteristics, expectations, and needs^(9,10-12)^. Therefore, before applying the TAM, it is essential to understand the core concepts of the model. The TAM posits that perceived ease of use (PEOU), and perceived usefulness (PU) are the two primary factors that influence an individual’s intention to use a technology, which, in turn, has an impact on their actual usage. In health care, that can be reflected in a patient searching for a new less-invasive procedure or for referring physicians to ask for an IR consult, for example. This, of course, is also for patients for whom IR procedures are indicated and who may benefit from them. Patients should make such decisions in consultation with their physician and in accordance with guideline recommendations.

Applying the TAM to medical innovation starts with adaptation of the TAM framework to the specific context of the innovation. It starts with identification of a specific technology or innovation to analyze and how potential users perceive its ease of use and usefulness.

The first step is to identify the target audience; that is, to determine the key stakeholders involved in the adoption of the medical innovation. This could include health care professionals, patients, caregivers, administrators, and other relevant parties. To apply the TAM, there is a need to collect data to assess the PEOU and PU, conducting surveys or interviews to gather those data from your target audience, using questions such as those listed in Tables 1 and 2. Health care providers play a pivotal role in the successful implementation of medical innovations and must be targeted. In contemporary health care, patients are increasingly regarded as active participants in their own health care decisions. This perspective has gained prominence in the era of patient-centered care. The TAM can also be applied to comprehend patient attitudes toward medical innovations, because their acceptance and engagement are vital components of the overall success of health care innovations. Little et al.^(13)^, in the GENESIS trial, used questionnaires to evaluate the usefulness of genicular artery embolization for patients.

**Table 1:** Examples of questions for technology acceptance model surveys.

**QUESTIONS FOR HEALTH CARE PROVIDERS**
How easy is it for you to use/refer a patient for this medical innovation in your daily work/practice?
To what extent do you believe this innovation will enhance patient care and treatment outcomes?
Do you think this medical innovation would enhance your ability to provide care or improve patient health?
How confident are you in using/recommending this innovation?
Do you think this innovation will increase risk for your patients?
**QUESTIONS FOR PATIENTS**
How easy do you find it to access this innovation for improvement of your health condition?
Do you think this medical innovation would enhance your health?
Are there any specific difficulties or challenges you encounter when facing this medical innovation?
Do you think there are more risks with this innovation than with the current treatment for your disease?
Do you believe this innovation will empower you to better manage your health and well-being?
In your view, will the use of this innovation lead to improved health outcomes and a better quality of life?

**Table 2:** Use of the questions of Table 1 to create information, using thyroid ablation for thyroid nodules as an example.

**QUESTIONS FOR HEALTH CARE PROVIDERS**
In which situations do you believe thyroid ablation will be indicated for your patients?
How confident are you in recommending this treatment?
Do you think thyroid ablation will increase risk for your patients? Which specific risks do you believe may increase?
Do you think thyroid ablation will decrease risk for your patients? Which specific risks do you believe may decrease?
To what extent do you believe thyroid ablation will improve outcomes of thyroid disease?
Do you think thyroid ablation would enhance your ability to treat thyroid nodules?
Given that it is a minimally invasive procedure, do you think more patients could be treated using the thyroid ablation technique?
**QUESTIONS FOR PATIENTS**
Has your doctor included thyroid ablation among the possible procedures to treat your thyroid disease?
How easy do you find it to access thyroid ablation for the treatment of your thyroid disease?
Do you think thyroid ablation may help enhance your health? In what way?
Are there any specific difficulties or challenges you encounter when facing the decision to seek out thyroid ablation?
Do you think your medical provider participated in your decision to look for thyroid ablation?
Do you think there is more risk associated with thyroid ablation than with thyroid surgery/radioactive iodine therapy? Which specific risks?
Do you believe thyroid ablation will help you feel better and live better?
In your view, will thyroid ablation improve health outcomes and quality of life in patients with diseases similar to yours?

### Identifying barriers and facilitators

On the basis of the TAM analysis, the key barriers to and facilitators of the adoption of the medical innovation can be identified. The barriers are often linked to factors such as a lack of communication skills, cultural differences, lack of trust in the physician, and limited knowledge of new technologies. Among the facilitators, a good physician–patient relationship is one of the most important^(14)^. This information will be valuable in developing strategies to promote adoption of a new technology.

### Developing intervention strategies

If the TAM analysis reveals barriers to acceptance, targeted strategies to address those issues should be developed. That could involve improving training and education, as well as enhancing the user interface of the technology and demonstrating the benefits of the innovation.

#### Pilot testing

Before widespread implementation of the medical innovation, consider conducting pilot tests to validate the effectiveness of your intervention strategies and gather feedback from referring physicians and the community.

#### Iteration and improvement

Continuously collect feedback and data to refine your strategies and improve the acceptance of the medical innovation. Feedback from patients offers valuable insights into perceptions of health care services, including medical innovation, among patients and service users alike. A thorough review of this feedback provides a direct understanding of the strengths and areas for improvement in care delivery and in the use of new technologies^(15)^. The TAM can be used as an ongoing assessment tool to measure changes in user perceptions over time.

#### Monitoring and evaluation

After the medical innovation is implemented, continue to monitor and evaluate its acceptance and usage. Use the TAM to periodically assess the ongoing impact that the technology has on health care practices.

The Greek Organization for the Health Care Provision created the Personal Health Insurance Record (PHIR), which is a service that enables authorized citizens to access all types of health services provided to them by the organization and submit and track reimbursement requests. The results of the study demonstrated that patients were satisfied with using the PHIR and will continue using it. In addition, PU and PEOU were highlighted as the main factors that affected the intention to use the PHIR, thus showing that the TAM is effective for evaluating digital health services^(16)^.

Regarding the TPB, it is important to acknowledge some of its well-established limitations. Although the model is widely used to predict behavioral intention, a discrepancy may exist between stated intention and actual behavior, which is often influenced by external and environmental factors beyond the control of the individual. Recognizing these limitations allows for a more accurate interpretation of results derived from TPB-based studies.

With respect to the TAM, the literature has evolved to include modern extensions, such as the TAM2 and the unified theory of acceptance and use of technology (UTAUT) model^(17)^. These frameworks expand the original constructs and integrate additional determinants of technology adoption, providing greater explanatory power. In the health care context, variables such as privacy concerns, data security, and regulatory requirements are particularly relevant, because they may significantly shape both intention and behavior regarding the acceptance of new technologies^(17,18)^.

Finally, although the TPB and TAM are traditionally presented as independent models, recent theoretical advances highlight the value of their integration. Contemporary frameworks, such as the UTAUT, synthesize constructs from both models, thereby offering a more comprehensive and practical tool for understanding behavioral intention and technology acceptance. This integration enables the development of unified instruments and interventions that are better suited to the complexity of health care environments^(17,18)^.

It is worth noting that the questionnaires in this paper are examples of suggested questions that can be used by the physician to understand their market and thus create a questionnaire. Each procedure or new therapy will require a specific questionnaire, and studies should therefore be conducted to validate them.

It is crucial to acknowledge the limitations of this study, which include the lack of more robust empirical validation for the application of the TPB and TAM specifically within IR. In addition, the reliance on online questionnaires may have introduced a sampling bias, thus limiting the representativeness of the findings. Finally, the scope of this research did not account for crucial systemic barriers to adherence, such as procedure costs, access to services, and insurance coverage, which warrant future investigation for a more comprehensive understanding.

There is currently a lack of concrete data in the literature regarding the proportion of patients who have knowledge about IR procedures or similar topics. This gap highlights the need for further in-depth studies to provide a more rigorous and comprehensive analysis of this subject. There is also a need for studies analyzing specific IR procedures, such as prostate artery embolization and cancer ablation.

## CONCLUSION

The TPB and TAM are instrumental frameworks in medical innovation, offering a systematic approach to evaluating and promoting the acceptance of new technologies and practices. As health care continues to evolve, with rapid technological advancements, application of these models may be pivotal in facilitating the successful integration of medical innovations, enhancing patient care, and optimizing health care practices. More studies are needed in order to validate behavioral instruments specific to IR.

## Data Availability

Not applicable

## References

[1] Huffman J, Nichols WK, Bath J (2021). Current hybrid interventions in vascular surgery: merging past
and present. Mo Med.

[2] Han A, Ahn S, Min SK (2019). Oncovascular surgery: essential roles of vascular surgeons in
cancer surgery. Vasc Specialist Int.

[3] Kelly MP, Barker M (2016). Why is changing health-related behaviour so
difficult?. Public Health.

[4] Barley E, Lawson V (2016). Using health psychology to help patients: theories of behaviour
change. Br J Nurs.

[5] Ajzen I (1991). The theory of planned behavior. Organ Behav Hum Decis Process.

[6] Davis FD (1989). Perceived usefulness, perceived ease of use, and user acceptance
of information technology. MIS Q.

[7] Glanz K, Bishop DB (2010). The role of behavioral science theory in development and
implementation of public health interventions. Annu Rev Public Health.

[8] Safi S, Thiessen T, Schmailzl KJ (2018). Acceptance and resistance of new digital technologies in
medicine: qualitative study. JMIR Res Protoc.

[9] Darker CD, French DP, Eves FF, Sniehotta FF (2010). An intervention to promote walking amongst the general population
based on an ‘extended’ theory of planned behaviour: a waiting list
randomised controlled trial. Psychol Health.

[10] Plotnikoff RC, Lubans DR, Costigan SA, McCargar L (2013). A test of the theory of planned behavior to predict physical
activity in an overweight/obese population sample of adolescents from
Alberta, Canada. Health Educ Behav.

[11] Dequanter S, Fobelets M, Steenhout I (2022). Determinants of technology adoption and continued use among
cognitively impaired older adults: a qualitative study. BMC Geriatr.

[12] Lee C, Coughlin JF (2015). Older adults’ adoption of technology: an integrated approach to
identifying determinants and barriers. J Prod Innov Manag.

[13] Little MW, Gibson M, Briggs J (2021). Genicular artery embolization in patients with osteoarthritis of
the knee (GENESIS) using permanent microspheres: interim
analysis. Cardiovasc Intervent Radiol.

[14] Deng Q, Zheng Y, Lu J, Zeng Z, Liu W (2021). What factors predict physicians’ utilization behavior of
contrast-enhanced ultrasound? Evidence from the integration of the Theory of
Planned Behavior and Technology Acceptance Model using a structural equation
modeling approach. BMC Med Inform Decis Mak.

[15] Hardavella G, Aamli-Gaagnat A, Saad N, Rousalova I, Sreter KB (2017). How to give and receive feedback effectively. Breathe (Sheff).

[16] Akritidi D, Gallos P, Koufi V, Malamateniou F (2022). Using an extended technology acceptance model to evaluate digital
health services. Stud Health Technol Inform.

[17] Lee AT, Ramasamy RK, Subbarao A. (2025). Understanding psychosocial barriers to healthcare technology
adoption: a review of TAM Technology Acceptance Model and Unified Theory of
Acceptance and Use of Technology and UTAUT frameworks. Healthcare (Basel).

[18] Bile Hassan I, Murad MAA, El-Shekeil I, Liu J (2022). Extending the UTAUT2 model with a privacy calculus model to
enhance the adoption of a health information application in
Malaysia. Informatics.

